# The ‘Nigerian Diet’ and Its Evolution: Review of the Existing Literature and Household Survey Data

**DOI:** 10.3390/foods12030443

**Published:** 2023-01-17

**Authors:** Ivica Petrikova, Ranjana Bhattacharjee, Paul D. Fraser

**Affiliations:** 1 Department of Politics, International Relations and Philosophy, Royal Holloway University of London, Egham TW20 0EX, UK; 2 International Institute of Tropical Agriculture, Ibadan 200001, Nigeria; 3 Department of Biological Sciences, Royal Holloway University of London, Egham TW20 0EX, UK

**Keywords:** diet, Nigeria, Nigerian diet, dietary transition, Living Standard Measurement Survey, Oyo

## Abstract

Natural and social science studies have commonly referenced a ‘typical’ or ‘habitual’ Nigerian diet, without defining what such a diet entails. Our study, based on a systematic review of the existing literature and an analysis of household-level survey data, describes the general outline of a common Nigerian diet and how it varies based on spatial, demographic, and socio-economic characteristics. We further try to establish whether Nigeria has embarked on a dietary transition common in most modern economies, marked by a greater consumption of processed foods, fats, and sugar at the expense of traditional whole cereals and pulses. We conclude that while a traditional Nigerian diet is still relatively healthy from an international perspective, it has indeed been transitioning, with an increasing inclusion of high-energy, high-fat, and high-sugar processed foods and a related growing incidence of overweight, obesity, and diet-related non-communicable diseases.

## 1. Introduction

The term ‘typical’ or ‘habitual Nigerian’ diet has long been used in biology, medicine, and public health, as well as in other studies [1,2,3,4,5,6,7]. However, there has been little research conducted into what foods the ‘typical Nigerian diet’ includes, how the diet differs in population subgroups, and how it has evolved over time. The lack of research on these topics in Nigeria is emblematic of a wider relative ignorance of the African continent by mainstream nutrition science and public health research [8]. However, it constitutes an omission critical not only to natural and social science studies referring to the term ‘Nigerian diet’ without properly defining it, but also for policy makers in Nigeria making decisions about the country’s public health without a good evidence base concerning what the country’s dietary patterns are and how they are changing.

Our study contributes to filling this research gap. We summarise which foods commonly form part of the diets of the Nigerian people and how these patterns vary across different Nigerian regions, rural and urban areas, and socio-economic strata. Furthermore, we examine how what Nigerians eat has changed over the past decade, to assess to what extent Nigeria has been undergoing the ‘nutrition transition’, characterised by an increased consumption of energy-dense foods (animal-sourced foods, plant oils, and sugars) at the expense of traditional whole cereals and pulses [9], accompanied by an increased incidence of non-communicable diseases (NCDs). We do so through a systematic review of the existing relevant literature and an analysis of household-level empirical data on food consumption from the World Bank’s Living Standard Measurement Surveys in 2010 and 2018 and our own survey conducted around Ibadan in Oyo State in 2021 (these are described in more detail in the Section 2). 

Since we do not have access to individual-level data on food consumption, our study does not provide precise nutritional information for individual diets; the study is, however, still indicative of Nigerian food consumption patterns across different spaces, socio-economic groups, and time. Furthermore, our findings provide important evidence to assess the degree to which Nigeria has been afflicted by the ‘triple burden of malnutrition’, a situation where countries experience simultaneously significant rates of underweight, overweight, and micronutrient deficiencies, which has been often exacerbated by the nutrition transition.

## 2. Materials and Methods

**Figure A1:**
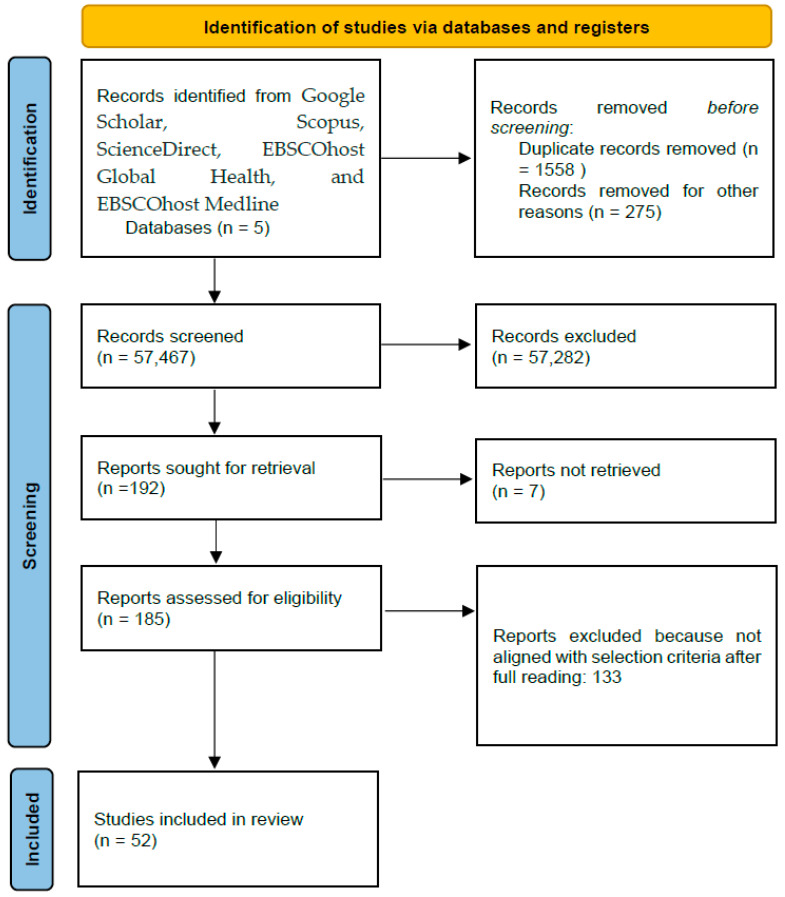
PRISMA flow diagram for identification of relevant articles for systematic review.

We adopted a two-pronged methodological approach. First, we conducted a systematic review of the literature to identify academic sources that refer to and describe the Nigerian diet and its evolution. We used the Preferred Reporting Items for Systematic Reviews and Meta-Analyses (PRISMA) 2020 guidelines [10]. Our PRISMA flow diagram is included in the Appendix (Figure A1). The study was registered with OSF Registries and is further explained in Section 2.1, Section 2.2 and Section 2.3.

Second, we complemented information from the systematic review with primary data on Nigerian people’s food consumption from the World Bank’s nationally representative Living Standard Measurement Surveys (LSMS) conducted in 2010 [11] and in 2018 [12] and with data from a survey [13] conducted in Oyo State by the International Institute of Tropical Agriculture (IITA), Nigeria, in collaboration with Royal Holloway University of London between May and September 2021, among 50 schoolchildren and their households (we further refer to this survey as the IITA survey). 

### 2.1. Sources and Search Strategy for Systematic Review of Literature

To find relevant academic articles, we searched several scientific databases, namely Google Scholar, Scopus, ScienceDirect, EBSCOhost Global Health, and EBSCOhost Medline. We used the following search terms: Nigerian diet, Nigerian diets, Nigeria dietary transition, diets in Nigeria, Nigerian food, and Nigeria food consumption, along with several similar variations. The titles and abstracts of the articles retrieved initially were examined for relevance against selection criteria described in greater detail in the next section. We read selected articles in their entirety and assessed them for inclusion. We further looked through the bibliography of included articles to identify further appropriate research.

### 2.2. Literature Selection Criteria

Table 1 displays the inclusion and exclusion criteria used to identify relevant research. We used a wide temporal timeframe, considering sources published between 1950 and May 2021 in the English language. We excluded articles that discussed the Nigerian diet(s) without specifying at all what foods such a diet contained. 

### 2.3. Extraction of Data from the Literature and Synthesis

From each relevant source identified from the literature in the systematic review, we extracted information about what foods were commonly consumed as part of the Nigerian diet, how these dietary patterns differed by population subgroups (regions, urban/rural areas, wealth categories, and gender), and how they changed over time. The text coding and survey data analysis were conducted by one researcher (IP), with methods verified by the other two researchers (RB and PDF).

### 2.4. Survey Data

The LSMS [11,12] surveyed a nationally representative sample of 5000 Nigerian households in 2010 and 2018, twice each year, once in the post-planting and once in the post-harvest period. The survey question to which we paid most attention in our study asked households—usually the head of the household—which foods, from a list of about 100 different foods, they had consumed at home in the seven days preceding the survey (all the food items are listed in tables in the Appendix). We aggregated the food item data into broader food groups and compared household averages across similar categories to those utilised in the analysis of data extracted from the existing literature: time (2018 versus 2010), different areas (urban versus rural and across Nigeria’s six zones: North-Central, North-East, North-West, South-East, South-South, and South-West), and wealth (by household wealth quartiles estimated through principal component analysis of household asset ownership data reported in the survey). 

We further supplemented our findings with an analysis of the data from the IITA survey [13]. For this survey, five different schools around Ibadan, Oyo State were selected, and from each school, ten children between the ages of 10 and 15 were surveyed along with their households. Table A2 and Table A3 in the Appendix display further summary statistics collected about the children. The main question of interest for our study in the survey asked the children and their households which food groups they had consumed in the two days preceding the survey. As a result of the small sample size and close geographical location of survey respondents, results from the IITA survey are only disaggregated in line with their households’ wealth quartiles, estimated, as in the LSMS surveys, through principal component analysis of household asset ownership data. 

## 3. Results

### 3.1. Typical Nigerian Diet

The general agreement in the literature has been that a traditional Nigerian diet was high in carbohydrates and fibre, low-to-moderate in fats, and relatively low in protein [1,2,5,6,7,14,15,16,17,18,19]. The 2018 LSMS survey [12], which enquired from more than 5000 households across the country what foods they had consumed in the preceding week, suggested that Nigerian reliance on carbohydrates for calories has continued, even though it is slightly below the sub-Saharan African average of 71%, at about 69% of daily energy intake per capita [20]. However, this is significantly above the averages in high-income countries, which in 2013 were estimated to be 52% in the UK, 49% in the US, and 46% in France, for example [21]. The carbohydrate sources consumed the most across Nigeria, according to the 2018 LSMS [12], were different types of grains, with almost all families surveyed having eaten them in the previous seven days (Table 2). More households consumed unrefined grains than refined ones, but the difference between the two in 2018 was significantly lower than in 2010. The consumption of starchy tubers such as cassava was also high, at almost 80% (Table 2). All schoolchildren and their families in the IITA survey also reported to have eaten grains or tubers in the two days before the survey (Table A3).

In terms of fat and protein consumption, research [20] has found fats to comprise slightly more (22%) and protein slightly less (9.3%) of the daily energy intake of Nigerians compared to the mean sub-Saharan African diet (19% and 9.8%, respectively). In contrast, an average inhabitant in the UK, as an example of a high-income country, obtained 36% of daily calories from fats and 12% from protein sources in 2013 [21]. The Nigerian diet can thus still be described as comparatively low in protein and moderate in fat intake from an international perspective.

Looking at the key sources of these macronutrients, in the 2018 LSMS [12] survey, 95% of Nigerian households regularly consumed fats and oils, with palm oil (91%) and groundnut oil (57%) being the most popular (Table A4). Most households also reported to have eaten a variety of protein sources, with ‘nuts and pulses’ (85%) and ‘fish and shellfish’ (70%) the most represented. A total of 60% of households consumed some meat in the week preceding the survey as well. In contrast, 4% of households ate no high-protein foods, such as pulses and nuts, fish and shellfish, meat, eggs, or dairy, although they likely consumed protein as part of their vegetable and tuber intake. As Thompson and Kelly [22] have noted, vegetables have traditionally been the cheapest and most readily available source of protein across Africa. Dairy and particularly eggs, as it appears from the 2018 LSMS [12] survey, have thus far remained relatively less popular in Nigeria, with only 48% and 26% households, respectively, having consumed any at home in the week before the survey (Table 2). Consumption of foods from animal sources among schoolchildren in the IITA survey [13] was comparatively higher, with 84% reporting to have eaten meat, 68% dairy, and 64% eggs over the 48-h period examined (Table A3). 

Taking a closer look at the specific foods eaten by Nigerian households, rice was the most frequently domestically consumed source of carbohydrates in 2018, followed by bread, garri (pounded cassava), sorghum, and maize (Table A4). Yams were the most ubiquitous in the ‘roots and tubers’ category, while white beans, brown beans, and groundnuts were most common in ‘nuts and pulses.’ In the IITA survey, households [13] also reported rice, garri, yam flour, and semolina as their most frequently consumed carbohydrate sources. From foods from animal sources, beef was eaten most frequently, followed by fish and chicken, in both the LSMS [11,12] and IITA survey respondents [13]. Onions, peppers, tomatoes, and okra were the most popular vegetables, with the consumption of eggplants and green leafy vegetables, such as jute mallow, water leaf, bitter leaf, cocoyam, and spinach leaves, also widespread. The most consumed fruits included watermelon, oranges and tangerines, mangoes, bananas, apples, pawpaw (papaya), coconuts, pineapples, and guava. These findings largely align with those of Alade [23], who also added to the list of popular Nigerian fruits grapefruits, pears, cashew fruits, and avocado pears. Additionally, common Nigerian vegetables include turnip and mustard greens, kale, mushrooms, pumpkins, collard greens, carrots, cabbage, and lettuce [23].

The specific composition of Nigerian meals varies significantly by region; however, one can generalise that it typically consists of a grain or starchy vegetable, accompanied by a ‘sauce’, also referred to as ‘soup’ or ‘stew’. The grain or starchy vegetable portion of the dish can be made up of rice, often either boiled or jollof (cooked with tomatoes, onions, and spices) [14]. ‘Swallows’, i.e., pounded or pureed grains or starchy vegetables that can be made into balls and swallowed without chewing, are also popular [14]. These differ across the country but can be made of yam (iya and amala isu), cassava (fufu, garri, eba and amala lafun), plantains (amala/elubo ogede), cocoyam (empkang nkuwo), corn flour (eko and agidi), wheat flour (semovita/semolina), fermented sorghum, maize, or millet (ogi and akamu), and many others [13,23]. 

The ‘sauce’ or ‘soup’ is a stew made of multiple ingredients, often including onions, tomatoes, peppers, and other vegetables, along with palm or another oil, meat, fish or crayfish, water, salt, and other seasonings [1,13,14,23]. Examples include ewedu soup (stew with jute mallow leaves and locust beans), afang soup (stew with spinach and water leaves), edikang ikong (stew with fluted pumpkin and water leaves), pepper soup, egusi soup (stew with ground melon seeds), efo riro (spinach stew with peppers and locust beans), miyan kuka (stew with dried baobab leaves), okra soup, and many others [1,4,23,24,25]. Fried foods, such as pof-pof (doughnuts), fried bean cakes (akara), and fried plantain (dodo), have also been traditionally popular, particularly as snacks [13]. 

The existing literature on the Nigerian diet has emphasised some of its aspects as positive and some as negative. A review of dietary quality in 187 countries between 1990 and 2010 [26] found the Nigerian diet to be quite healthy from an international perspective. In the consumption of healthy food items (fruits, vegetables, beans and legumes, nuts and seeds, whole grains, milk, total polyunsaturated fatty acids, fish, plant omega-3s, and dietary fibre), Nigeria ranked 35th out of 187 countries (19th percentile), and in the non-consumption of seven unhealthy items (unprocessed red meats, processed meats, sugar-sweetened beverages, saturated fat, trans fat, dietary cholesterol, and sodium), it ranked 40th out of 187 countries (21st percentile). A review of dietary health consequences in 195 countries between 1995 and 2017 [27] meanwhile concluded that in adults aged 25 years and older, Nigerians experienced the lowest proportion of age-standardised diet-related deaths (11%) and disability-adjusted life years (7%) out of all the countries in the sample. Other studies have highlighted the protective effects of the traditional high-fibre and relatively low-fat diet against colon cancer [7], high cholesterol levels [28], and diabetes [29]. The nutritional benefits of Nigeria’s traditionally consumed green leafy vegetables have also been widely extolled [22,29,30,31]. 

On the less positive side, some concerns have been raised regarding vitamin A intake through the typical Nigerian diet, given that about 16% of children have been recently estimated to be vitamin A deficient [32]. However, others [25] have pointed out that the strand of Nigerian diet that relies on high consumption of green leafy vegetables and palm oils could provide the population with enough beta-carotene (main source of vitamin A in Nigeria). 

Further apprehension about the typical Nigerian diet has revolved around the diet’s relatively high reliance on carbohydrates for energy and frequently low dietary diversity [33]. Some scholars have highlighted the consequently higher risks of nutritional and particularly protein and micronutrient deficiencies, potentially leading to arrested bone growth [5,17]. This issue has been particularly notable among young children, who are often weaned on cereal pap, with little intake of other food groups [34,35,36]. This has contributed to a situation where more than 37% of Nigeria’s children under five years old are stunted (too short for their age) [37]. A less prevalent, but still serious, issue is one of rickets (Ref. [38] – estimated in 2012 at 1.2%), which is generally not connected with vitamin D deficiency (as Nigerian children tend to spend a lot of time outdoors in the sunlight) but rather with low intakes of calcium due to a relative scarcity of dairy in the typical Nigerian diet (Ref. [39], see also Table 2). Finally, the Nigerian diet has also been estimated to be high in phytates, at three times the typical intake in a UK or a US diet, which has been linked with reduced absorption of iron, calcium, and other minerals [40]. 

### 3.2. Differences in Nigerian Diet across Different Characteristics

The ‘typical Nigerian diet’ discussed so far varies quite widely; however, based on both Nigeria’s geography and on individual and household characteristics. This section explores first the differences by the rural-urban divide and the country’s six regions and second by individual/household characteristics of gender and wealth. 

#### 3.2.1. Differences by Area

Data from the 2018 LSMS survey [12] on food groups consumed by households in urban versus rural Nigeria show that the dietary diversity of urban households is higher. This is in line with previous findings [16]. Urban families in the dataset consumed almost all the food groups assessed more frequently, except for sweets and alcohol (Table 3). Urban consumption exceeded rural consumption particularly in animal-sourced foods (see also [18]) and vitamin A-rich fruits and vegetables. 

A more detailed look at the data reveals that the consumption of traditional or indigenous foods was higher in rural than in urban areas (Table A5). Rural families ate more sorghum, millet, maize, cassava, white beans, groundnuts, and kola nuts. They also drank more fresh milk and ate more dried fish and dried vegetables. On the other hand, urban households consumed significantly more rice, yams, bread and wheat flour, plantains, brown beans, fruits (particularly bananas, tangerines, pineapples, watermelons, and apples), tomatoes, beef, chicken, eggs, milk powder, and instant chocolate drinks (such as Milo, a Nestle product). Data from the existing literature align with these survey findings, suggesting that urban Nigerian families rely more on cereal consumption for their daily energy intake than rural families [15] and consume more ready-to-eat foods including cornflakes, other breakfast cereals, and instant noodles (e.g., Indomie) [36].

Considering the geographical differences in food consumption, Table 4 shows the commonly consumed food groups in Nigeria’s six zones: North-Central (where Abuja, the capital of Nigeria, is located), North-East, North-West, South-East, South-South, and South-West (where Lagos, Nigeria’s largest city, is located). The clearest distinction is between diets in the northern compared to the southern states, with southern states exhibiting much higher levels of dietary diversity. This was also observed in research that examined Nigeria’s three different agro-ecological zones (the dry savannah, moist savannah, and tropical forest (from north to south)), that noted a high incidence of low dietary diversity particularly in the dry savannah in northern Nigeria [33]. 

While in all Nigerian regions, most people consumed grains regularly, Table A6 suggests that the type differs considerably by geographical area. Sorghum, millet, and maize flour dominate in the north, whereas rice, wheat flour, and garri (pounded cassava) are much more prevalent in the south. These differences broadly relate to divergent agricultural patterns in the country’s distinct agro-ecological zones. While in the drier savannah in the north, cereals such as millet, wheat, and sorghum/guinea corn are cultivated, moist savannahs and tropical forests are more hospitable to the cultivation of legumes, tubers, and root crops such as yam and cassava [13,23,35].

Nutrient-dense foods, including foods from animal sources, green leafy vegetables, and fruits, are overall more abundant in Nigeria’s south than north (Table A6). Looking at the remarkable differences in the consumption in meat, mutton is eaten only in the North-West and North-East, while bush meat (the consumption of which has become a subject of some international controversy since the COVID-19 pandemic) is consumed more in the south. Fresh milk is drunk almost solely in northern states but people in southern states consume more reconstituted milk powder. From fruits and vegetables, pineapples, paw paws, apples, eggplants, and tomatoes are consumed significantly more in the south. Fresh fish is eaten most in the South-South zone, which has the longest coastline of the six Nigerian geographical areas. In northern Nigeria, people eat more sugar and drink more tea, but drink less alcohol than people in the south. From specific dishes, many of the ‘soups’ mentioned earlier, including the edikang ikong, egusi, ewedu, and efo riro, are typically consumed in the south [13]. Meanwhile, dishes such as miyan kuka (soup with baobab leaves), tuwo shinkafa (rice balls), tuwo dawa (ground sorghum and cassava), and suya (grilled meats) are more popular in the north [23,41]. 

The existing literature discussing the regional differences in Nigerian diets has also reported a higher incidence of diet-related non-communicable diseases in Nigeria’s southern zones. For example, while the prevalence of hypercholesterolaemia is only 4% in the North-East, it is 54% in the South-South region [28]. South-Eastern Nigeria also exhibits higher diabetes rates than the rest of the country, at 3.7% compared to the average of 1.9% in the whole of Nigeria [29]. This fact has been attributed to the consumption of higher calorie staple foods in the south, such as yams, cassava, garri, and soups high in salt. Obesity in the South-East is at 9.7%, also higher than the national average (5.3%). This higher incidence may be also related to the traditional veneration of obesity in parts of the region as emblematic of status and power [41].

#### 3.2.2. Differences by Individual/Household Characteristics (Wealth/Income Groups, Gender, Ethnic Groups)

The differences in food consumption trends between different wealth groups in Nigeria mirror to some extent those between rural and urban areas. The LSMS [12] data on food group consumption, divided by the four wealth groups (one least wealthy, four wealthiest – groups estimated on the basis of household asset ownership) is displayed in Table 5, and shows that the wealthiest quartile of households consumed all food groups more frequently than the least wealthy quartile. However, the difference was significantly larger for some food groups than others. For example, almost twice the percentage of the top quartile than of the bottom one ate vitamin A-rich fruits, meat, and dairy products in the week prior to the survey. The same was true vis à vis the consumption of processed food items, such as soft and stimulant drinks (coffee, tea, and hot chocolate). The largest difference was in the consumption of eggs, which was three times higher in the wealthiest quartile than in the least wealthy one. 

The same pattern emerged from the IITA survey data [13]. Children in the wealthiest quartile, as shown in Table 6, consumed flesh foods one-and-a-half times more and eggs and dairy two times more frequently than children from the least wealthy quartile. Children from better-off households also ate notably more salty snacks and drank more soft drinks, fruit juice, and caffeinated drinks than children from poorer households.

Looking at specific foods (Table A7), wealthier households ate significantly more refined grain products, including bread, biscuits, and yellow garri, than poorer households, as well as more plantains, bananas, oranges, watermelon, beef, chicken, and eggs. They also drank more reconstituted milk, chocolate drinks, and tea. However, less wealthy households did consume some foods more frequently, notably traditional cereals such as sorghum and millet, dried vegetables, kola nuts, fresh milk, and pito (traditional alcohol from fermented millet or sorghum). 

The literature to date has commented only a little on how typical Nigerian diets vary by wealth. One existing observation has been of higher dietary diversity among wealthier groups [16], which aligns with our findings from the LSMS [11,12] and IITA [13] surveys. Higher consumption of a more diverse range of foods, including more ‘convenient’ or processed food, has also had some negative effects alongside positive ones, however, such as a higher incidence of obesity and hypercholesterolaemia among Nigeria’s wealthier strata [28]. The IITA survey data (Table 7) have indicated that it is in the middle wealth groups where one might find the healthiest Nigerian diets currently, with a higher consumption of nutrient-dense foods than the lowest wealth groups but a lower consumption of processed foods than the wealthiest groups. (We classified legumes, dairy, flesh foods, eggs, leafy greens, vitamin A-rich fruits and vegetables, and other fruits and vegetables as healthy foods and salty snacks, fried snacks, sweets, soft drinks, and caffeinated drinks as unhealthy foods.)

Interestingly, similar observations regarding wealth groups have been made in the existing literature with regard to Nigerian women versus men, i.e., that Nigerian women suffer relatively more from obesity and high cholesterol levels than Nigerian men [28]. The underlying reason for this difference is less clear than for the difference among wealth groups. It appears to arise in childhood, with Nigerian girls significantly less likely to be underweight and stunted than Nigerian boys and more likely to be overweight [42]. This may be partially caused by different weaning and feeding of male and female children. While the most recent Demographic Health Survey [37] in Nigeria showed little statistical difference in the food groups consumed by male and female children under five years old, the IITA survey conducted among older children showed that Nigerian tween and teenage girls ate more unhealthy and fewer healthy foods than boys (Table 7). The greater rate of being overweight among girls and women could be also related to different levels of physical activity, with both female children [42] and female adults [13] reporting to exercise less than males. 

### 3.3. Changes over Time

Similar to all diets, the Nigerian diet has been evolving. Researchers have observed that diets worldwide have become more homogeneous over the past 50 years, evolving along trajectory of the ‘nutrition transition’ [43]. This transition generally involves a declining consumption of traditional and unrefined cereals, pulses, fruits, and vegetables and an increased consumption of energy-dense foods, particularly foods from animal sources, plant oils, and sugars [43,44,45]. Such dietary changes are often accompanied by increased incidence of overweight, obesity, and diet-related NCDs including hypertension and diabetes [45]. Many lower-income countries have begun the nutrition transition when a substantial portion of their population still suffers from undernourishment and micronutrient deficiencies; therefore, they face the so-called ‘double’ or even ‘triple burden of malnutrition’ [46,47]. The following section assesses to what extent the Nigerian diet has changed in recent decades and whether these changes align with the hallmarks of the ‘nutrition transition’.

One aspect of the nutrition transition that evidently has occurred in Nigeria has been a shift in consumption away from traditional grains such as sorghum and millet towards rice and maize [48]. As Table A4 shows, while 24% Nigerian households consumed millet in the week before the 2010 LSMS, only 18% did so in 2018. Conversely, the prevalence of rice consumption increased from 86% to 92% of households between 2010 and 2018. There was also a significant corresponding increase in the consumption of bread, from 54% to 67%, and of maize flour, used for making breakfast pap, fufu, tuwo masara, corn pudding, etc., from 6% to 24% of households. 

Other commonly noted features of the nutrition transition include higher consumption of foods from animal sources and altered patterns of fruit and vegetable consumption [43]. The first of these two can observations also be observed in the Nigerian LSMS data. While the overall level of meat and fish consumption remained similar between 2010 and 2018, the proportion of households reporting to have eaten eggs and dairy products increased from 13% and 38%, respectively, in 2010 to 26% and 48% in 2018. These average figures may hide some regional differences. Glew et al. [49], for example, found that northern Fulani communities in rural areas consumed significantly more daily protein in the form of meat and milk than Fulani communities in urban areas, indicating that urbanisation may have reduced the consumption of foods form animal sources in some communities. However, overall, Nigerians are now consuming significantly more protein from animal sources than they did in the past. 

Trends in the consumption of fruits and vegetables are harder to decipher from the available data. The existing literature has claimed that the nutrition transition commonly leads to a decline in the consumption of indigenous fruits and vegetables [50,51] or even of all fruits and vegetables [52]. Support for the first claim, i.e., the reduction in Nigerians eating traditional fruits and vegetables, cannot easily be discerned from the LSMS data, as the 2010 food frequency questionnaire was not as detailed in recording specific fruits and vegetables as the 2018 one. However, as explained earlier, the consumption of traditional cereals in Nigeria has declined (Table A4). Meanwhile, other research has shown that the diversification of local diets in most places has been accompanied by a worldwide homogenisation of diets, with agricultural production around the world focussing more on the cultivation of major world crops [43]. It is hence likely that the consumption of less internationally known indigenous fruits and vegetables has declined in Nigeria as well. Nevertheless, the LSMS data do not show Nigerians eating fewer fruits and vegetables altogether. In fact, it is quite the opposite; the data suggest that in the eight years between the two analysed surveys [11,12], the consumption of particularly vitamin A-rich fruits and vegetables and green leafy vegetables increased. Results from the IITA survey [11] indicate that it is particularly in the Nigerian economic middle classes where this increase has occurred.

Another common characteristic of the nutrition transition is an increase in the consumption of energy-dense and processed foods. Existing research from Africa generally and from Nigeria specifically confirms the existence of this trend [53,54]. It has been linked to the rising prevalence of fast-food restaurants in Nigeria, including Mr Biggs, Chicken Republic, Nando’s, Kentucky Fried Chicken, Domino’s Pizza, and McDonald’s [55,56]. These eateries generally serve food high in sugar, salt, and saturated fats, accompanied by bottled high-sugar soft drinks or canned and packaged fruit juices [55]. The meals are seen as both tasty and affordable, with eating out consequently increasing among all socio-economic groups [55].

The LSMS data do not demonstrate a large increase in the home consumption of processed foods in Nigeria between 2010 and 2018, except for refined grains (particularly in the form of bread) and instant chocolate drinks, but that is likely because the 2010 survey did not inquire about the consumption of many processed foods. The data do indicate, however, that over the course of the decade, Nigerian families began to eat out more frequently (Table 8). While the proportion of households eating full meals away from home did not change significantly, the prevalence of purchasing snacks (e.g., sandwiches, biscuits, meat pies, donuts, pof-pof, and akara) and roasted or boiled starchy vegetables (potatoes, corn, plantains, and yam) when outside increased significantly, from 33% to 47% and from 17% to 25%, respectively. The consumption of soft drinks outside the home rose even more, from 27% of households purchasing them in the week before the survey in 2010 to 42% of households in 2018 (more than a 50% increase). Schoolchildren and their parents in the IITA survey [13] similarly reported to have increased the frequency with which they purchased lunches or snacks away from home over time. Furthermore, children from wealthier households in the IITA survey [13] demonstrated a growing popularity of eating convenience foods for breakfast and on the weekends. While children from poorer households often consumed rice or spaghetti and stew with beans, meat or fish for breakfast, children from better-off households were more likely to eat toast, porridge, or breakfast cereal with milk, cocoa, or tea. On the weekends, wealthier children attested to frequently eating snacks such as yam or sweet potatoes with fried eggs, eggy bread, bean cakes (moin-moin or akara), and mashed potatoes.

**Figure 1:**
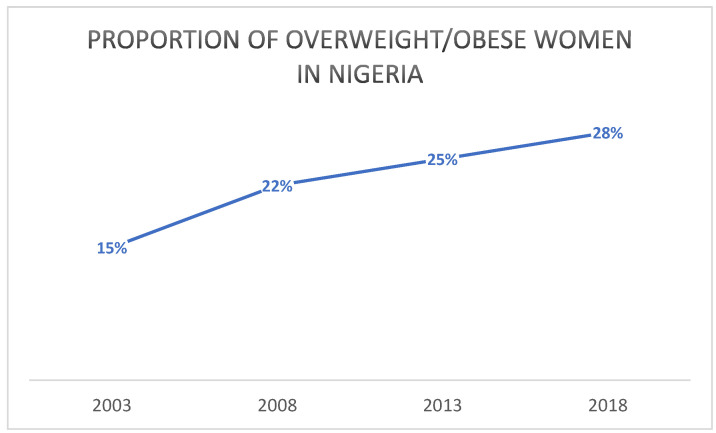
Proportion of overweight/obese Nigerian women between 2003 and 2018. Source: DHS 2018.

Due to the higher consumption of processed foods, countries undergoing the nutrition transition commonly experience growing incidence of obesity and diet-related NCDs, including hypercholesterolaemia, hypertension, and diabetes. As mentioned already, the prevalence of these health problems has begun to rise in Nigeria as well in recent decades, and particularly quickly in more affluent areas and households [57]. Adeloye et al. [58] estimated the proportion of Nigerian overweight adults in 2021 at 25%, with women slightly more often overweight (25.5%) than men (25.2%). The equivalent percentage for overweight women obtained from the DHS surveys (Figure 1) was similar, with 28% of women found to be heavier than the healthy weight in 2018. This constitutes almost a doubling from 2003, when 15% of women were estimated to be overweight. The prevalence of overweight was significantly higher in the southern regions—40% in the South-East, 43% in the South-South, and 38% in the South-West—than in the northern regions, where it was 26% in the North-Central, 15% in the North-East, and 16% in the North-West [37]. The South-South region has commensurately seen the highest prevalence of hypercholesterolaemia in Nigeria (50.4%) [36], while the South-East the highest prevalence of diabetes, at 3.5% [29]. 

The growing popularity of processed foods and fast-food outlets across the world has been explained by a combination of increasing urbanisation and a related search for convenience and tastiness [53,59,60]. However, it has also been argued that the consumption of ‘Western-style foods’ in lower-income, non-Western countries is seen as a proxy for wealth and/or status, which further increases the popularity of such foods [61]. All these factors are likely at play in driving forward Nigeria’s nutrition transition. 

## 4. Discussion and Conclusions

Our review of what constitutes a typical ‘Nigerian’ diet, how the diet varies by different characteristics, and how it has changed over time has demonstrated that while the traditional Nigerian diet was quite healthy from an international perspective, the transition that the diet is currently undergoing is gradually reducing its positive nutritional qualities. 

Studies from various disciplines have used the term ‘Nigerian diet’ without explicitly defining what such a diet entails. A review of the existing literature, combined with an analysis of the World Bank’s Living Standard Measurement Surveys from 2010 [11] and 2018 [12] and of our own survey of 50 schoolchildren and their respective households from Oyo State (IITA survey [13]), have all shown that the typical Nigerian diet is relatively low in protein, high in carbohydrates and fibre, and moderate in the intake of fats and oils. In terms of carbohydrate sources, Nigerian meals have typically relied on cereals including rice, sorghum, millet, and maize and on roots and tubers such as cassava and yams. While this is still broadly true, over time the globally most important crops such as rice, maize, and wheat have become more popular in Nigeria and traditional crops such as sorghum and millet have become less popular. This trend was most notable in urban areas, southern parts of the country, and among wealthier households. The shift was also accompanied by an increasing consumption of refined grains, including bread, biscuits, and donuts. Nevertheless, mashed or pounded cereals, roots, or tubers, i.e., ‘swallows’, have thus far remained a key component of Nigerian main meals in all parts of the country. 

The swallows are typically served with ‘soups’ or ‘sauces’ that include fats, vegetables, water, spices, flavourings/seasonings (such as Maggi cubes), and also frequently protein sources such as pulses, meat (primarily beef and/or chicken), fish, or seafood. The most consumed vegetables are onions, peppers, tomatoes, okra, and various green leaves (spinach, jute mallow, cocoyam, water leaf, bitter leaf, etc.). The average consumption of vegetables in Nigeria is high by international standards and has been increasing over time. The same appears to be true of fruit consumption, which involves predominantly tropical fruits such as paw paws, bananas, oranges and tangerines, pineapples, watermelon, mangoes, coconuts, and guava. However, the consumption of temperate climate fruits (e.g., apples and pears) has also increased over time, particularly among wealthier urban households. Finally, while the consumption of foods from animal sources and associated animal proteins remains relatively low in Nigeria from an international perspective, it has increased over time, especially in the form of eggs and dairy products. 

The nutrition transition in Nigeria has apparently already led to an increase in the average consumption of foods from animal sources, vegetables, and fruits. It has also brought about higher dietary diversity, and this has been generally linked with lower malnutrition rates [62]. However, whether these dietary changes have helped reduce child malnutrition rates in Nigeria is not clear. While the rate of underweight children under five years old declined in the country from 29% in 2003 to 22% in 2018, the reduction in stunting rates was only from 38% percent in 2003 to 37% in 2018 [37]. 

It is conversely obvious that the nutrition transition has had some negative health consequences for the Nigerian population. Both reports in the literature and the LSMS and IITA survey data reveal that Nigerians have begun to eat more meals away from home over the last few decades, and that the popularity of fast-food restaurants selling high fat, high salt, and/or high sugar processed foods is on the rise. This trend has contributed to the rising incidence of overweight and obesity in the population, with the prevalence of overweight adult Nigerian women doubling between 2003 and 2018. The prevalence of diet-related non-communicable conditions and diseases such as hypertension, hypercholesterolaemia, and diabetes has risen commensurately. 

The nutrition transition has by now affected almost all countries in the world, bringing about an increasing homogenisation of the world’s countries’ diets [43]. It has entailed an increased consumption of energy-dense foods (foods from animal sources, plant oils, and sugars) at the expense of traditional whole cereals and pulses, which has resulted in a rising incidence of diet-related non-communicable diseases. This article has argued that the ‘typical’ Nigerian diet was traditionally healthy in international comparisons, and even recently, Afshin et al. [27] concluded that in adults aged 25 years and older, Nigeria experienced the lowest proportion of age-standardised diet-related deaths and disability-adjusted life years lost out of 195 countries examined over the preceding two decades. Nevertheless, more recent research and empirical data indicate that the average Nigerian diet is changing and that these changes have negative public health implications. It is a crucial, but not easy, task for the Nigerian government, as it is for the governments of most Western countries, to try to figure out ways in which the positive aspects of the nutrition transition could be retained while the negative aspects are eradicated or at least ameliorated, to address the growing threat of the ‘triple burden of malnutrition’. Relevant policies could involve, for example, combining the provision of cash transfers or healthy free school meals with nutrition education and/or with a nation-wide roll out of community and school gardens [63].

## Figures and Tables

**Table 1:** Inclusion and exclusion criteria.

Inclusion Criteria	Exclusion Criteria
Publication period: January 1950–May 2021	Articles referring to the Nigerian diet without defining it in any way
Language: English	Articles examining chemical components of a specific food consumed in Nigeria without reference to the wider composition of Nigerian diets
Articles describing as their main focus or side focus the common components of diets/food consumption in Nigeria as a whole or a in a segment of Nigerian society (region, state, specific area, children, farmers, etc.)	Articles related to nutrition outcomes in Nigeria that do not specify any foods/food groups typically consumed by the study population
Study designs: all	

**Table 2:** Food groups eaten by Nigerian households in in the 7 days prior to the surveys in 2018 and 2010 [11,12].

Food Groups	2018	2010
Grains	97%	97%
Unrefined	96%	93%
Refined	86%	66%
Potatoes, cassava, other tubers	78%	75%
Pumpkin, carrots, squash (and other orange vegetables)	88%	90%
Dark green leafy vegetables	30%	9%
Mangos, papayas, other Vitamin A-rich fruits	45%	18%
Other fruits and vegetables	96%	92%
Beans, peas, lentils, other pulses and nuts	85%	78%
Eggs	26%	13%
Meat (pork, beef, lamb, chicken, other)	60%	61%
Fish or shelfish	70%	72%
Dairy	48%	38%
Fats and oils	95%	92%
Spices	56%	50%
Sweets and sweeteners	58%	55%
Bottled water	35%	24%
Soft drinks	27%	28%
Tea, coffee, hot chocolate, and kola nut	50%	26%
Alcohol	9%	12%

**Table 3:** Food groups eaten by urban and rural Nigerian households in the 7 days prior to the survey [12].

	Urban	Rural
Grains	98%	98%
Unrefined	97%	96%
Refined	91%	85%
Potatoes, cassava, other tubers	89%	74%
Pumpkin, carrots, squash (and other orange vegetables)	95%	87%
Dark green leafy vegetables	32%	29%
Mangos, papayas, other Vitamin A-rich fruits	55%	40%
Other fruits and vegetables	97%	96%
Beans, peas, lentils, other pulses and nuts	87%	85%
Eggs	38%	20%
Meat (pork, beef, lamb, chicken, other)	68%	56%
Fish or shelfish	80%	67%
Dairy	60%	43%
Fats and oils	97%	95%
Salt	96%	96%
Spices	60%	54%
Sweets and sweeteners	57%	59%
Bottled water	56%	26%
Soft drinks	30%	26%
Tea, coffee, hot chocolate, and kola nut	61%	45%
Alcohol	7%	10%

**Table 4:** Food groups eaten by Nigerian households in different regions in the 7 days prior to the survey [12].

	NC	NE	NW	SE	SS	SW
Grains	99%	99%	99%	99%	96%	96%
Unrefined	99%	93%	98%	98%	93%	96%
Refined	91%	82%	81%	91%	84%	91%
Potatoes, cassava, other tubers	87%	41%	55%	97%	99%	97%
Pumpkin, carrots, squash (and other orange vegetables)	88%	76%	85%	97%	94%	96%
Dark green leafy vegetables	30%	29%	14%	26%	46%	39%
Mangos, papayas, other Vitamin A-rich fruits	31%	34%	50%	51%	53%	52%
Other fruits and vegetables	97%	95%	92%	99%	99%	97%
Beans, peas, lentils, other pulses and nuts	85%	82%	83%	90%	91%	82%
Eggs	18%	8%	22%	25%	40%	43%
Meat (pork, beef, lamb, chicken, other)	54%	60%	57%	71%	66%	56%
Fish or shelfish	76%	44%	34%	93%	94%	87%
Cheese, yoghurt, other milk products	38%	24%	54%	66%	58%	51%
Fats and oils	95%	93%	94%	98%	98%	97%
Salt	95%	95%	95%	98%	96%	96%
Spices	53%	30%	39%	85%	85%	56%
Sweets and sweeteners	61%	78%	74%	54%	42%	41%
Bottled water	27%	19%	30%	41%	47%	51%
Soft drinks	20%	13%	22%	51%	39%	20%
Tea, coffee, hot chocolate, and kola nut	48%	41%	45%	65%	58%	55%
Alcohol	8%	6%	2%	18%	17%	6%

Lowest numbers for regions are highlighted in red, the highest are in green.

**Table 5:** Food groups eaten by Nigerian households across different wealth groups in the 7 days prior to the survey (I poorest–IV richest) [12].

Wealth Group	I	II	III	IV
Grains	95%	97%	99%	98%
Unrefined	92%	96%	98%	98%
Refined	79%	86%	90%	92%
Potatoes, cassava, other tubers	71%	75%	81%	87%
Pumpkin, carrots, squash (and other orange vegetables)	80%	88%	91%	95%
Dark green leafy vegetables	30%	29%	28%	34%
Mangos, papayas, other Vitamin A-rich fruits	31%	41%	51%	57%
Other fruits and vegetables	92%	96%	97%	97%
Beans, peas, lentils, other pulses and nuts	76%	84%	88%	92%
Eggs	13%	22%	28%	39%
Meat (pork, beef, lamb, chicken, other)	41%	55%	67%	77%
Fish or shelfish	65%	67%	71%	78%
Cheese, yoghurt, other milk products	32%	45%	53%	63%
Fats and oils	91%	95%	97%	97%
Salt	92%	95%	96%	96%
Spices	48%	53%	58%	65%
Sweets and sweeteners	44%	58%	64%	66%
Bottled water	21%	31%	40%	50%
Soft drinks	18%	25%	29%	38%
Tea, coffee, hot chocolate, and kola nut	35%	45%	55%	65%
Alcohol	10%	8%	8%	10%

**Table 6:** Food groups consumed by children in Oyo State across different wealth groups in the 2 days prior to the survey (I poorest–IV richest) [13].

	I	II	III	IV
Grains, tubers	100%	100%	100%	100%
Orange vegetables and fruits (pumpkin, carrots, orange, etc.)	62%	50%	77%	50%
Dark green leafy vegetables	77%	100%	85%	83%
Other fruits and vegetables	54%	83%	92%	83%
Pulses and nuts	62%	83%	62%	75%
Eggs	38%	75%	62%	83%
Flesh foods (pork, beef, lamb, chicken, fish, shelfish etc.)	69%	67%	100%	100%
Milk, cheese, yoghurt, other dairy products	46%	50%	85%	92%
Fats and oils	100%	100%	100%	100%
Sweets and sweeteners	85%	100%	92%	75%
Salty snacks (crisps, popcorn, fried snacks)	38%	67%	54%	92%
Soft drinks	31%	58%	69%	42%
Fruit juice	8%	8%	15%	75%
Tea, coffee, hot chocolate, and kola nut	38%	75%	85%	75%

**Table 7:** Average number of healthy and unhealthy food groups consumed in the 2 days prior to the survey by children in Oyo State across different wealth quartiles and gender [13].

	Healthy Foods	Unhealthy Foods
**Wealth quartiles**		
I	3.4	1.8
II	5.6	3.0
III	5.7	3.1
IV	5.7	3.8
**Gender**		
Male children	5.5	2.8
Female children	4.7	3.0

**Table 8:** Meals eaten out of home in the 7 days prior to the survey in 2018 versus 2010 [11,12].

Eating Out in the Past 7 Days	2018	2010
1. Breakfast (full meal)	27%	27%
2. Lunch (full meal)	32%	33%
3. Dinner (full meal)	9%	10%
4. Side dishes (pepper soup, nkwobi, suya, isiewu, asun)	18%	17%
5. Snacks (sandwiches, biscuits, meatpies, donuts, pofpof, akara)	47%	33%
6. Dairy based beverages (milk, yoghurt, fura)	15%	14%
7. Vegetables and roasted and boiled items (carrot, pears, boiled/roasted corn, roasted plantain, sugar cane, roasted yam etc.)	25%	17%
8. Non alcoholic drinks (Coke, Fanta, zobo, kunu)	42%	27%
9. Alcoholic drinks (palm wine, beer etc.)	14%	12%
Total	25%	21%

## Data Availability

Most of the study drew on existing literature and publicly available datasets (LSMS and DHS). The IITA survey data is available through Figshare.

## Appendix A

**Table A1:** Demographic characteristics of surveyed children [13].

		Mean	Min	Max
Child age		12.92	10	16
Child school grade		7.14	5	10
Child gender	*Female*	0.48	0	1
	*Male*	0.52	0	1
HH ethnicity	*Igbo*	0.80	0	1
	*Other*	0.20	0	1
HH religion	*Christian*	0.58	0	1
	*Muslim*	0.42	0	1
HH size		6.29	3	15

**Table A2:** Wealth characteristics of surveyed children [13].

	Mean	Min	Max
Access to flushing toilet	0.72	0	1
Water pipe in hh	0.18	0	1
Fridge	0.48	0	1
Freezer	0.42	0	1
Washing machine	0.22	0	1
TV	0.78	0	1
Phone	0.78	0	1
Fan	0.84	0	1
AC	0.14	0	1
Generator	0.42	0	1
Bicycle	0.18	0	1
Motorcycle	0.22	0	1
Car	0.50	0	1

**Table A3:** Food groups consumed by Oyo children in the 2 days prior to the survey [13].

Grains, Tubers	100%
Orange vegetables and fruits (pumpkin, carrots, orange, etc.)	60%
Dark green leafy vegetables	86%
Other fruits and vegetables	78%
Pulses and nuts	70%
Eggs	64%
Flesh foods (pork, beef, lamb, chicken, fish, shelfish etc.)	84%
Milk, cheese, yoghurt, other dairy products	68%
Fats and oils	100%
Sweets and sweeteners	88%
Salty snacks (crisps, popcorn, fried snacks)	62%
Soft drinks	50%
Fruit juice	26%
Tea, coffee, hot chocolate, and kola nut	68%

**Table A4:** Foods consumed in the 7 days prior to the survey by Nigerian households in 2010 and 2018 [11,12].

Item	2018	2010
Guinea corn/sorghum	30%	31%
Millet	18%	24%
Rice	92%	86%
Maize flour	24%	6%
Yam flour	9%	9%
Cassava flour	17%	12%
Wheat flour	6%	3%
Maize	26%	32%
Other grains and flour	1%	2%
Bread	67%	54%
Cake	2%	
Buns/Pofpof/Donuts	7%	
Biscuits	22%	
Meat Pie/Sausage Roll	3%	
Cassava-roots	13%	15%
Yam-roots	45%	50%
Gari-white	39%	32%
Gari-yellow	25%	19%
Cocoyam	10%	8%
Plantains	26%	19%
Sweet potatoes	16%	10%
Potatoes	4%	3%
Other roots and tuber	0%	1%
Soya beans	4%	3%
Brown beans	18%	23%
White beans	63%	51%
Groundnuts	35%	19%
Other nuts/seeds/pulses	0%	7%
Coconut	8%	
Kola nut	14%	
Cashew nut	0%	
Palm oil	91%	80%
Butter/Margarine	1%	6%
Groundnuts Oil	57%	42%
Other oil and Fat	1%	5%
Animal fat	0%	
Bananas	23%	17%
Orange/tangerine	26%	14%
Mangoes	1%	4%
Avocado pear	2%	2%
Pineapples	7%	6%
Guava	6%	
Other fruits	0%	3%
Pawpaw	16%	
Watermelon	18%	
Apples	4%	
Tomatoes	65%	65%
Tomato puree (canned)	25%	25%
Onions	91%	79%
Garden eggs/egg plant	13%	12%
Okra	59%	54%
Pepper	85%	73%
Leaves (cocoyam, spinach)	30%	9%
Other vegetables	2%	21%
Chicken	14%	10%
Duck	0%	1%
Other domestic poultry	0%	1%
Agricultural eggs	21%	10%
Local eggs	5%	2%
Other eggs (not chicken)	0%	1%
Beef	43%	47%
Mutton	2%	5%
Pork	1%	2%
Goat	9%	7%
Wild game/bush meat	2%	2%
Other meat (excl. poultry)	0%	1%
Fish-fresh	15%	15%
Fish-frozen	32%	37%
Fish-smoked	18%	19%
Fish-dried	27%	26%
Snails	2%	3%
Seafood	5%	3%
Canned fish/seafood	0%	1%
Other fish or seafood	1%	1%
Fresh milk	7%	9%
Milk powder	35%	16%
Baby milk powder	2%	3%
Milk tinned (unsweetened)	7%	13%
Cheese (wara)	4%	
Other milk products	0%	2%
Coffee	1%	1%
Chocolate drinks (Milo)	29%	15%
Tea	14%	13%
Sugar	57%	49%
Honey	3%	4%
Other sweets/confectionary	0%	2%
Salt	96%	50%
Unground Ogbono	11%	
Ground Ogbono	7%	
Ground Pepper	16%	
Melon	20%	
Melon (ground)	12%	
Other Spices	20%	
Bottled water	4%	4%
Sachet water	35%	21%
Malt drinks	14%	14%
Soft drinks	19%	20%
Fruit juice	1%	2%
Other non-alcoholic drinks	0%	1%
Beer (local and imported)	5%	7%
Palm wine	4%	4%
Pito	1%	1%
Gin	1%	1%
Other alcoholic beverages	0%	1%

**Table A5:** Foods consumed in the 7 days prior to the survey by urban and rural Nigerian households [12].

Item	Urban	Rural
Guinea corn/sorghum	11%	38%
Millet	10%	22%
Rice	99%	90%
Maize flour	17%	28%
Yam flour	16%	5%
Cassava flour	18%	16%
Wheat flour	13%	3%
Maize	18%	30%
Other grains and flour	3%	1%
Bread	78%	62%
Cake	3%	2%
Buns/Pofpof/Donuts	7%	7%
Biscuits	22%	22%
Meat Pie/Sausage Roll	4%	2%
Cassava-roots	4%	18%
Yam-roots	56%	40%
Gari-white	52%	33%
Gari-yellow	26%	24%
Cocoyam	10%	9%
Plantains	36%	22%
Sweet potatoes	14%	17%
Potatoes	5%	3%
Soya beans	3%	5%
Brown beans	32%	11%
White beans	54%	68%
Groundnuts	28%	38%
Coconut	6%	9%
Kola nut	9%	16%
Palm oil	93%	90%
Butter/Margarine	3%	1%
Groundnuts Oil	65%	54%
Bananas	28%	21%
Orange/tangerine	35%	22%
Mangoes	1%	1%
Avocado pear	2%	1%
Pineapples	12%	5%
Guava	3%	7%
Pawpaw	15%	16%
Watermelon	27%	14%
Apples	10%	2%
Tomatoes	82%	58%
Tomato puree (canned)	30%	23%
Onions	93%	90%
Garden eggs/egg plant	14%	12%
Okra	52%	64%
Peppers	93%	83%
Leaves (Cocoyam, Spinach, etc	32%	30%
Other vegetables (fresh or canned)	1%	2%
Chicken	18%	12%
Agricultural eggs	35%	15%
Local eggs	3%	6%
Beef	55%	38%
Mutton	1%	2%
Pork	1%	2%
Goat	7%	10%
Wild game/bush meat	1%	3%
Fish-fresh	13%	16%
Fish-frozen	48%	24%
Fish-smoked	20%	17%
Fish-dried	22%	30%
Snails	2%	2%
Seafood (lobster, crab, prawns, etc)	5%	6%
Fresh milk	3%	8%
Milk powder	49%	28%
Baby milk powder	2%	1%
Milk tinned (unsweetened)	11%	5%
Cheese (wara)	2%	5%
Coffee	2%	1%
Chocolate drinks (including Milo)	41%	23%
Tea	19%	12%
Sugar	54%	58%
Honey	5%	2%
Salt	96%	95%
Unground Ogbono	11%	11%
Ground Ogbono	8%	6%
Ground Pepper	17%	15%
Melon	25%	19%
Melon (ground)	14%	11%
Other Spices	18%	21%
Bottled water	8%	2%
Sachet water	54%	25%
Malt drinks	16%	13%
Soft drinks (Coca Cola, Sprite...)	21%	18%
Fruit juice canned/Pack	2%	1%
Other non-alcoholic drinks	0%	0%
Beer (local and imported)	4%	5%
Palm wine	2%	5%
Pito	0%	1%
Gin	1%	2%
Other alcoholic beverages	0%	0%

**Table A6:** Foods consumed in the 7 days prior to the survey by Nigerian households in different zones [12].

	NC	NE	NW	SE	SS	SW
Guinea corn/sorghum	51%	51%	69%	1%	2%	3%
Millet	16%	29%	59%	1%	0%	1%
Rice	95%	77%	87%	100%	100%	98%
Maize flour	42%	54%	37%	5%	1%	6%
Yam flour	14%	0%	0%	0%	1%	35%
Cassava flour	36%	7%	5%	12%	5%	34%
Wheat flour	4%	3%	8%	3%	4%	15%
Maize	25%	26%	33%	35%	22%	17%
Other grains and flour	1%	0%	0%	1%	1%	5%
Bread	54%	54%	60%	79%	77%	80%
Cake	1%	1%	3%	3%	2%	1%
Buns/Pofpof/Donuts	4%	2%	6%	15%	11%	3%
Biscuits	16%	11%	20%	42%	36%	7%
Meat Pie/Sausage Roll	1%	0%	3%	7%	6%	1%
Cassava - roots	6%	11%	17%	27%	18%	1%
Yam-roots	54%	19%	20%	63%	55%	61%
Gari-white	50%	9%	25%	36%	33%	79%
Gari-yellow	11%	3%	5%	52%	67%	11%
Cocoyam	1%	2%	4%	24%	17%	12%
Plantains	11%	1%	2%	42%	64%	40%
Sweet potatoes	10%	23%	34%	13%	10%	7%
Potatoes	4%	4%	5%	3%	3%	2%
Soya beans	4%	4%	10%	4%	1%	2%
Brown beans	13%	10%	3%	10%	18%	53%
White beans	69%	68%	77%	68%	66%	31%
Groundnuts	32%	26%	23%	58%	56%	15%
Coconut	2%	0%	2%	19%	23%	5%
Kola nut	8%	23%	26%	13%	6%	8%
Palm oil	92%	79%	86%	97%	97%	95%
Butter/Margarine	1%	0%	1%	1%	2%	3%
Groundnuts Oil	43%	60%	75%	62%	55%	49%
Animal fat	1%	1%	1%	0%	0%	0%
Bananas	17%	12%	13%	31%	37%	28%
Orange/tangerine	17%	26%	34%	18%	26%	37%
Mangoes	1%	1%	1%	1%	0%	1%
Avocado pear	1%	1%	0%	5%	3%	1%
Pineapples	4%	1%	4%	9%	15%	12%
Pawpaw	6%	1%	3%	36%	31%	17%
Watermelon	16%	14%	33%	14%	13%	19%
Guava	1%	2%	2%	21%	10%	1%
Apples	3%	1%	1%	6%	4%	12%
Tomatoes	72%	41%	66%	72%	52%	89%
Tomato puree (canned)	13%	2%	5%	62%	48%	20%
Onions	88%	88%	87%	97%	94%	93%
Garden eggs/egg plant	8%	5%	10%	32%	13%	9%
Okra	77%	70%	59%	50%	61%	41%
Peppers	85%	67%	73%	92%	100%	96%
Leaves (Cocoyam, Spinach, etc	30%	29%	14%	26%	46%	39%
Other vegetables (fresh or canned)	1%	1%	4%	1%	1%	1%
Chicken	12%	14%	15%	12%	15%	17%
Duck	1%	1%	0%	0%	0%	0%
Agricultural eggs	14%	4%	14%	22%	36%	38%
Local eggs	4%	3%	8%	4%	5%	5%
Beef	36%	39%	32%	62%	50%	42%
Mutton	1%	6%	5%	0%	0%	0%
Pork	3%	1%	0%	1%	3%	0%
Goat	8%	12%	17%	5%	11%	2%
Wild game/bush meat	2%	1%	0%	2%	5%	3%
Fish-fresh	14%	10%	17%	8%	32%	9%
Fish-frozen	27%	2%	10%	51%	42%	58%
Fish-smoked	12%	8%	5%	32%	25%	27%
Fish-dried	38%	26%	5%	43%	40%	12%
Snails	1%	0%	0%	3%	7%	2%
Seafood (lobster, crab, prawns, etc)	0%	0%	0%	9%	23%	1%
Other fish or seafood	0%	0%	0%	3%	4%	0%
Fresh milk	3%	10%	24%	0%	0%	1%
Milk powder	28%	12%	23%	58%	50%	39%
Baby milk powder	1%	0%	1%	2%	3%	2%
Milk tinned (unsweetened)	6%	1%	4%	11%	9%	13%
Cheese (wara)	6%	2%	12%	0%	0%	3%
Other milk products	0%	0%	2%	0%	0%	0%
Coffee	1%	0%	3%	2%	1%	1%
Chocolate drinks (including Milo)	23%	4%	8%	52%	46%	42%
Tea	10%	23%	18%	10%	12%	13%
Sugar	60%	77%	74%	53%	40%	38%
Honey	2%	2%	4%	2%	4%	5%
Salt	95%	95%	95%	98%	96%	95%
Unground Ogbono	5%	0%	0%	35%	22%	4%
Ground Ogbono	6%	0%	1%	10%	19%	5%
Ground Pepper	13%	6%	11%	21%	32%	10%
Melon	11%	1%	2%	49%	38%	22%
Melon (ground)	20%	1%	2%	9%	22%	16%
Other Spices	15%	26%	17%	17%	23%	22%
Bottled water	1%	1%	2%	4%	4%	9%
Sachet water	26%	19%	29%	40%	46%	48%
Malt drinks	8%	6%	8%	34%	19%	8%
Soft drinks (Coca Cola, Sprite...)	14%	8%	19%	30%	29%	15%
Fruit juice canned/Pack	1%	0%	1%	2%	1%	1%
Beer (local and imported)	4%	3%	0%	11%	8%	2%
Palm wine	2%	1%	1%	9%	7%	3%
Pito	2%	1%	0%	0%	0%	0%
Gin	1%	0%	0%	2%	4%	1%
Other alcoholic beverages	0%	1%	0%	0%	0%	0%

Lowest numbers for regions are highlighted in red, the highest are in green.

**Table A7:** Foods consumed in the 7 days prior to the survey by Nigerian households across different wealth groups [12].

Wealth Group	I	II	III	IV
Guinea corn/sorghum	33%	36%	28%	21%
Millet	17%	22%	17%	14%
Rice	82%	90%	97%	100%
Maize flour	26%	26%	23%	22%
Yam flour	7%	8%	9%	10%
Cassava flour	19%	17%	15%	14%
Wheat flour	2%	4%	7%	11%
Maize	26%	26%	26%	26%
Other grains and flour	1%	1%	2%	2%
Bread	54%	64%	72%	80%
Cake	1%	2%	1%	4%
Buns/Pofpof/Donuts	5%	6%	8%	9%
Biscuits	15%	20%	25%	27%
Meat Pie/Sausage Roll	2%	2%	3%	5%
Cassava-roots	14%	13%	14%	13%
Yam-roots	38%	40%	47%	57%
Gari-white	36%	39%	40%	40%
Gari-yellow	18%	22%	26%	33%
Cocoyam	9%	9%	11%	11%
Plantains	17%	21%	29%	40%
Sweet potatoes	12%	17%	17%	18%
Potatoes	2%	3%	3%	6%
Soya beans	3%	5%	4%	4%
Brown beans	13%	15%	20%	23%
White beans	54%	65%	67%	69%
Groundnuts	33%	32%	35%	38%
Coconut	6%	6%	9%	12%
Kola nut	13%	15%	14%	13%
Palm oil	85%	91%	93%	95%
Butter/Margarine	0%	1%	1%	3%
Groundnuts Oil	40%	56%	65%	70%
Bananas	17%	19%	24%	32%
Orange/tangerine	18%	25%	30%	34%
Mangoes	1%	1%	1%	1%
Avocado pear	1%	1%	2%	3%
Pineapples	4%	5%	8%	13%
Guava	5%	4%	6%	8%
Pawpaw	13%	12%	17%	20%
Watermelon	9%	17%	22%	27%
Apples	1%	4%	5%	9%
Tomatoes	50%	64%	70%	78%
Tomato puree (canned)	17%	20%	27%	36%
Onions	85%	91%	94%	95%
Garden eggs/egg plant	8%	10%	14%	19%
Okra	57%	60%	60%	61%
Peppers	54%	63%	67%	74%
Leaves (Cocoyam, Spinach, etc	74%	84%	88%	95%
Chicken	8%	11%	15%	24%
Agricultural eggs	10%	17%	23%	36%
Local eggs	4%	5%	6%	5%
Beef	27%	38%	50%	60%
Mutton	1%	2%	2%	2%
Pork	2%	2%	1%	2%
Goat	6%	9%	10%	12%
Wild game/bush meat	2%	2%	2%	2%
Fish - fresh	13%	15%	14%	18%
Fish - frozen	25%	29%	34%	40%
Fish - smoked	16%	17%	18%	21%
Fish - dried	26%	24%	28%	31%
Snails	2%	2%	2%	3%
Seafood (lobster, crab, prawns, etc)	5%	5%	6%	6%
Fresh milk	7%	8%	7%	5%
Milk powder	20%	31%	39%	51%
Baby milk powder	1%	1%	2%	3%
Milk tinned (unsweetened)	4%	5%	8%	12%
Cheese (wara)	4%	5%	4%	3%
Coffee	0%	1%	1%	3%
Chocolate drinks (including Milo)	17%	24%	34%	42%
Tea	9%	12%	15%	21%
Sugar	44%	57%	62%	65%
Honey	1%	2%	4%	5%
Salt	93%	95%	97%	97%
Unground Ogbono	9%	9%	11%	16%
Ground Ogbono	4%	5%	7%	10%
Ground Pepper	13%	15%	16%	19%
Melon	16%	18%	23%	26%
Melon (ground)	9%	11%	13%	15%
Other Spices	19%	20%	19%	21%
Bottled water	1%	2%	4%	8%
Sachet water	21%	31%	39%	48%
Malt drinks	7%	11%	15%	22%
Soft drinks (Coca Cola, Sprite...)	13%	18%	21%	25%
Fruit juice canned/Pack	0%	1%	1%	2%
Beer (local and imported)	4%	4%	5%	6%
Palm wine	4%	3%	4%	4%
Pito	1%	1%	0%	0%
Gin	1%	1%	1%	1%

Lowest numbers for regions are highlighted in red, the highest are in green.
